# The paradox of progress: how RAE research may be fueling inequity in sport

**DOI:** 10.3389/fspor.2026.1746566

**Published:** 2026-05-29

**Authors:** Sean Horton, Kristy L. Smith, Laura Chittle, Jess C. Dixon

**Affiliations:** 1 Department of Kinesiology, University of Windsor, Windsor, ON, Canada; 2 Centre for Teaching and Learning, University of Windsor, Windsor, ON, Canada

**Keywords:** birth quartiles, physical maturity, relative age effects, selection bias, talent identification, youth development

## Introduction

Relative age effects (RAEs), first observed four decades ago and documented in countless studies since, describe selection biases that advantage children born earlier in an age-group cut-off period (e.g., January–March in many youth sport systems) over their younger peers born later in the same year (1, 2). Relatively greater chronological age can often manifest itself though greater size, strength, coordination, and/or cognitive development, and hence, greater perceived talent vs. younger peers, leading to a selection bias toward relatively older children. In time, performance-based selection leads to more developmental opportunities, better coaching, and higher rates of continued participation. These benefits compound over time, effectively stacking the deck in favor of older children within a given cohort. Age-based systems often fail in two important ways. First, they leave a considerable amount of talent “on the table” as relatively younger children are excluded or, alternatively, drop out before their abilities can be properly identified or developed (3). Second, there is an inherent inequity in systems that produce, and tolerate, such an outcome.

Recent articles by Barnsley (4) and Grondin (5) display the shared sense of frustration, even indignation, at the persistence of RAEs. Despite more than 40 years of research and repeated calls for reform, little has changed in the structural organization of the majority of youth sport systems in North America. Relative age effects remain a stubborn fixture—obvious, blatant, and unfair—yet their persistence exposes a troubling paradox; the more RAEs are publicized, the more they seem to be exploited rather than dismantled.

## Unintended consequences

Despite, or perhaps because of, the attempts of researchers (ourselves included) to mobilize knowledge beyond traditional academic outlets [e.g., (6–8)], anecdotal evidence abounds of parents acting in surprising, but not entirely unpredictable ways, given the literature on what has been called “intensive parenting” [e.g., (9, 10)]. We have heard numerous examples of parents, sometimes even researchers themselves, deliberately planning pregnancies so their children were born just after a cut-off date in an attempt to maximize relative age advantages. These examples provide eerie parallels to concerns we raised a decade ago (11) about parents “gaming the system” rather than advocating for fairer structures. Specifically, we presented evidence that Triple Crown breeders and/or owners intentionally schedule the births of thoroughbred horses in the early months of the calendar year to enhance their odds of racing success. This pattern of birth timing contrasts sharply with what would naturally occur in the wild (11). We went on to suggest that, as public awareness of RAEs increased, it was possible that parents would adopt similarly drastic strategies to secure advantages for their children in sports and education. An interaction at a recent sport conference revealed the unsettling immediacy with which some individuals grasp and act on this information. A participant who had just learned about RAEs eagerly devised a catchy diagram encouraging a) conception between April and June, for b) corresponding births between January and March, paired with the slogan: “Conceive, Believe, Achieve” and concluding with c) an image of a child triumphantly hoisting a trophy.

Such episodes recall Michael Lewis's reflections on his book *Liar's Poker* (12), which detailed his experience as a bond trader. This bestseller was, at least in part, an attempt to warn readers about the corruption and greed on Wall Street. Just as Lewis lamented the fact that he appeared to inspire more individuals to go into finance than he dissuaded (13), collectively as researchers we may have convinced more people to exploit the system than to change it.

Awareness, without accompanying policy shifts, may contribute to an arms race among parents seeking every possible edge for their children, including delaying their kindergarten enrollment in an attempt to gain academic, athletic, and social advantages (14). The consequences of this unintended mobilization are significant. Without meaningful reform from governing bodies, we risk entrenching and even exacerbating the inequities that RAEs produce. This may occur despite the fact that arbitrarily imposed cut-off dates can be changed suddenly [e.g., (15)], along with the alarming possibility that relatively older children, who benefit most from early opportunities, may also face higher rates of injury (16) and burnout (17). Moreover, those with relative age advantages at younger ages do not always have successful transitions from junior to senior levels and may ultimately be less successful at the professional level (18). Conversely, there exists the potential for an “underdog effect”, whereby later-born children — those who survive a system stacked against them — develop superior skills through the challenges that they encounter (18, 19). These are just some of the problems inherent in treating child rearing like horse breeding.

In our opinion, change must come from the top down: international and national sport organizations need to work with researchers and other stakeholders in order to evaluate, standardize, and implement best practices that recognize RAEs and mitigate their impact. Without such change, parents are likely to continue seeking advantages associated with relative age. Even when organizations attempt to address RAEs, they often do so with insufficient understanding or flawed strategies. For example, changing a cut-off date does nothing except change the cohort that derives the advantages and risks leaving parents confused about the nature of the change and its potential effectiveness, or lack thereof (15).

Clearly, more research on effective solutions is required. As Webdale and colleagues (20) noted in their systematic review of proposed solutions to RAEs, most remain theoretical, with few attempts at implementation. The authors, understandably, urge caution given the possibility of both positively and negatively affecting the career and life outcomes of the athletes involved. Furthermore, Towlson and colleagues (21) have emphasized the importance of accounting for and distinguishing between relative age and biological maturation; these are distinct entities, and potential solutions must account for both. There are, however, costs to inaction considering the negative effects already occurring within current systems, not least of which is the subset of children who are prematurely excluded.

While solutions remain underexplored, there are well-established working hypotheses regarding the mechanisms at play that inform viable strategies. Perhaps the most compelling is the Matthew Effect, whereby early advantages accumulate and compound over time [for a comprehensive discussion of the Matthew Effect, along with the related Galatea and Pygmalion effects, see Hancock et al. (22)]. One potentially effective means of countering the Matthew Effect is to delay the age at which children are selected, streamed, or specialized based on perceived talent. Support for this approach comes from recent evidence presented by Güllich et al. (23), who found that high performance at young ages, often driven by early specialization, was *negatively* correlated with the very highest levels of adult performance. The authors suggested that delaying specialization may be one important way of mitigating the risks associated with overtraining, burnout, and overuse injuries.

Norway's youth sport system has evolved in alignment with these principles, emphasizing broad participation, sport sampling, and the elimination of ability-based streaming and competitive leagues prior to age 13. A number of commentators have attributed Norway's sustained success in the Winter Olympics and in sports more broadly, despite its modest population size, to its philosophy on youth sport and associated policies [e.g., (24, 25)].

## Discussion/conclusion

So where does this leave us? Relative age effects are not simply an academic curiosity; they are deeply rooted structural inequities that impact countless children's physical, social, and psychological development. Sport organizations, leagues, and governing bodies must bear at least some of the responsibility for leading this reform. Without systemic action, the burden shifts unfairly to parents and children, who are left to either make do in an unjust system or seek to game it.

Forty years of research have laid bare the stubborn persistence of RAEs (4, 5). If an indictment is to be made, it should not be directed at parents and researchers alone but also at the organizations that have failed to translate overwhelming evidence into meaningful action and reform. Thus, we believe it is time for parents, researchers, and organizations to work together to turn decades of evidence into lasting change. Until they do, we risk perpetuating a cycle in which awareness breeds exploitation rather than equal opportunity, with our children, ironically, bearing the costs of our good intentions.
